# Supplementary material for: Microalgae and Bacteria Interaction—Evidence for Division of Diligence in the Alga Microbiota
Source: Microbiol Spectr. 2022 Aug 1;10(4):e00633-22. doi: 10.1128/spectrum.00633-22 (PMC9430724; doi:10.1128/spectrum.00633-22)

Supplemental FIGURES and TABLES

Supplemental FIGURE S1: Genome annotation and phylogenetic tree of *Dyadobacter* sp. HH091. A) circular graphical display of the distribution of the genome annotation of *Dyadobacter* sp. HH091. This includes, from outer to inner rings, the contigs, CDS on the forward strand, CDS on the reverse strand, RNA genes, CDS with homology to known antimicrobial resistance genes, CDS with homology to know virulence factors, GC content and GC skew. B) The colors of the CDS on the forward and reverse strand indicate the subsystem that these genes belong to (see Subsystems). C) The phylogenetic tree highlighting the position of *Dyadobacter* sp. HH091 relative to the type strains of other species within Bacteroidota. The phylogenetic tree was generated using the codon tree method within PATRIC, which used cross-genus families (PGFams) as homology groups [73]. 100 PGFams were found among these selected genomes using Codon Tree analysis, and the aligned proteins and coding DNA from single-copy genes were used for RAxML analysis. *Dyadobacter* sp. HH091 was included as an outgroup. FigTree was used for the tree visualization [74].

Supplemental FIGURE S2: FACS analyses of *Scenedesmus quadricauda* MZCH 10104 in co-culture with the strain Dyadobacter sp. HH091. A) algae + bacteria at the starting point of experiment, 5.56% of bacteria (I),13.2% of lysed algal cells (II), 59.4% of algae (III); B) algae + bacteria over a time period of 13 days, 3.49% of bacteria (I), 18.8% of lysed algal cells (II) and, 70.8% of healthy microalgae (III); C) axenic culture of algae without HH091, 3.24% of bacteria (I), 27.4% of lysed algal cells (II) and 57.7% of algae cells (III). FACs analyses demonstrated the improved fitness of *S. quadricauda* co-cultured with HH091.

Supplemental TABLE S1: Overall numbers of sequences and contigs generated for Genome analyses of *Dyadobacter* sp. HH091.

Supplemental TABLE S2: Predicted *Dyadobacter* sp. HH091 glycosyl hydrolases.

Supplemental TABLE S3: Predicted *Dyadobacter* sp. HH091 glycosyl hydrolases involved in microalgae polysaccharides digestion.

Supplemental TABLE S4: Predicted *Dyadobacter* sp. HH091 polysaccharide lyases.

Supplemental TABLE S5: Predicted *Dyadobacter* sp. HH091 carbohydrate esterases involved in polysaccharide utilization.

Supplemental TABLE S6: Overall numbers of sequences and contigs generated for the transcriptome datasets.

Supplemental TABLE S7: Protein family comparison of possible competitive and plant-bacteria interaction pathways across the dominant members of microbiome (*Porphyrobacter, Variovorax* and *Dyadobacter*) of *S. quadricauda* MZCH 10104. Numbers of proteins are marked with the following colors: ■ 0, ■ 1, ■ 2, and ■ 3+.

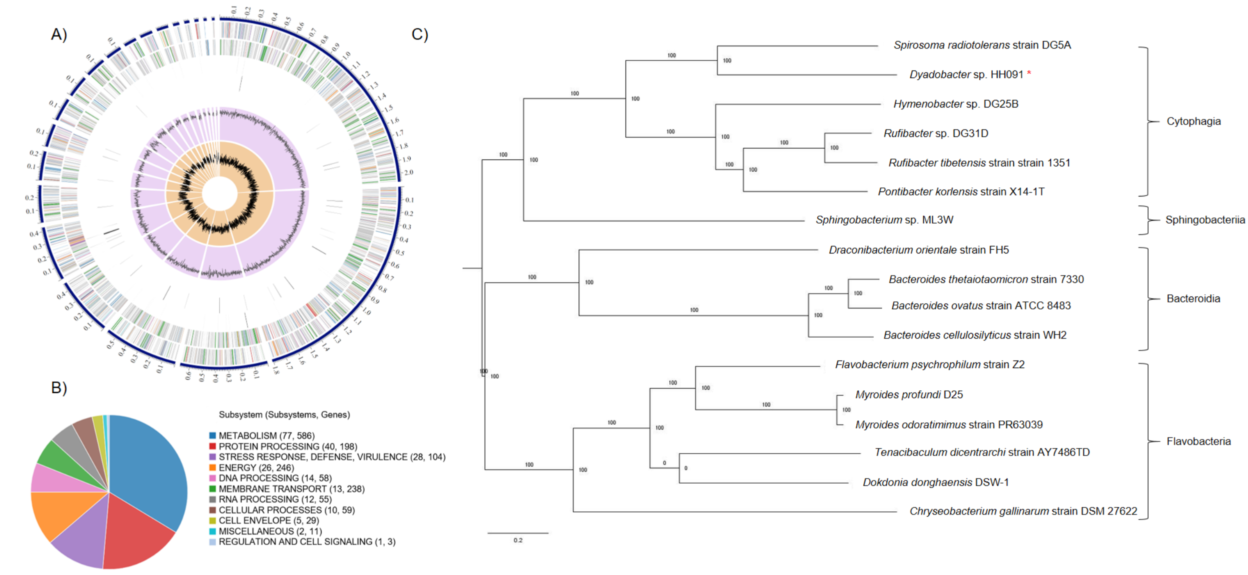

FIGURE S1: Genome annotation and phylogenetic tree of *Dyadobacter* sp. HH091. A) circular graphical display of the distribution of the genome annotation of *Dyadobacter* sp. HH091. This includes, from outer to inner rings, the contigs, CDS on the forward strand, CDS on the reverse strand, RNA genes, CDS with homology to known antimicrobial resistance genes, CDS with homology to know virulence factors, GC content and GC skew. B) The colors of the CDS on the forward and reverse strand indicate the subsystem that these genes belong to (see Subsystems). C) The phylogenetic tree highlighting the position of *Dyadobacter* sp. HH091 relative to the type strains of other species within Bacteroidota. The phylogenetic tree was generated using the codon tree method within PATRIC, which used cross-genus families (PGFams) as homology groups [73]. 100 PGFams were found among these selected genomes using Codon Tree analysis, and the aligned proteins and coding DNA from single-copy genes were used for RAxML analysis. *Dyadobacter* sp. HH091 was included as an outgroup. FigTree was used for the tree visualization [74].

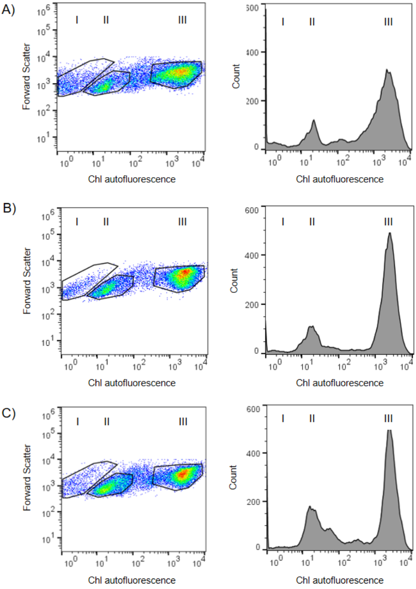

FIGURE S2: FACS analyses of *Scenedesmus quadricauda* MZCH 10104 in co-culture with the strain *Dyadobacter* sp. HH091. A) algae + bacteria at the starting point of experiment, 5.56% of bacteria (I),13.2% of lysed algal cells (II), 59.4% of algae (III); B) algae + bacteria over a time period of 13 days, 3.49% of bacteria (I), 18.8% of lysed algal cells (II) and, 70.8% of healthy microalgae (III); C) axenic culture of algae without HH091, 3.24% of bacteria (I), 27.4% of lysed algal cells (II) and 57.7% of algae cells (III). FACs analyses demonstrated the improved fitness of *S. quadricauda* co-cultured with HH091.

Supplemental TABLE S1: Overall numbers of sequences and contigs generated for Genome analyses of *Dyadobacter* sp. HH091.

	***Dyadobacter* sp. HH091**
**Contigs-assembly (Spades)**	
No.	80
Total length (bp)	7,862,706
No. ≥ 1000 bp	66
N50 size (bp)	607,803
Largest (bp)	1,195,963
GC (%)	43.81
**IMG ID genome statistic**	
IMG ID	222279
Number of bases	7,837,776
GC count [%]	43.87
Number of protein coding genes	6,565
% of assembled protein coding genes	99.05

Supplemental TABLE S2: Predicted *Dyadobacter* sp. HH091 glycosyl hydrolases[1].

Pfam	Glycosyl hydrolase family	Number of genes
pfam00232	Glycosyl hydrolase family 1	2
pfam00703	Glycosyl hydrolases family 2 (beta-glucuronidase)	4
pfam00933	Glycosyl hydrolase family 3	2
pfam00759	Glycosyl hydrolase family 9	2
pfam00331	Glycosyl hydrolase family 10 (xylanase)	1
pfam00723	Glycosyl hydrolases family 15	1
pfam00722	Glycosyl hydrolases family 16	3
pfam00704	Glycosyl hydrolases family 18	1
pfam00728	Glycosyl hydrolase family 20	6
pfam01183	Glycosyl hydrolases family 25	1
pfam01183	Glycosyl hydrolases family 25 (1,4-beta-N-acetylmuramidase)	1
pfam02156	Glycosyl hydrolase family 26 (mannan endo-1,4-beta-mannosidase)	2
pfam00295	Glycosyl hydrolases family 28	6
pfam02055	Glycosyl hydrolase family 30	1
pfam17189	Glycosyl hydrolase family 30	2
pfam01055	Glycosyl hydrolases family 31	3
pfam08244	Glycosyl hydrolases family 32	1
pfam17167	Glycosyl hydrolase family 36	1
pfam01229	Glycosyl hydrolases family 39	1
pfam02449	Glycosyl hydrolase family 42 (Beta-galactosidase)	4
pfam08532	Glycosyl hydrolase family 42M (Beta-galactosidase trimerisation domain)	1
pfam04616	Glycosyl hydrolases family 43 (beta-galactosidase, beta-xylosidase, alpha-N-arabinofuranosidase)	8
pfam12891	Glycoside hydrolase family 44	1
pfam03632	Glycosyl hydrolase family 65	1
pfam07477	Glycosyl hydrolase family 67	1
pfam02446	Glycosyl hydrolase family 77 (4-alpha-glucanotransferase)	1
pfam07470	Glycosyl Hydrolase Family 88	3

pfam07971	Glycosyl hydrolase family 92	2
pfam17132	Glycosyl hydrolase family 106 (alpha-L-rhamnosidase)	1
pfam07944	Glycosyl hydrolase family 127 (Beta-L-arabinofuranosidase)	3
pfam04041	Glycosyl hydrolase family 130 (beta-1,4-mannooligosaccharide phosphorylase)	2
Total		69

[1] Predicted functions were assigned by routines used for updating the Carbohydrate Active Enzymes database (http://www.cazy.org/).

Supplemental TABLE S3: Predicted *Dyadobacter* sp. HH091 glycosyl hydrolases involved in microalgae polysaccharides digestion[1].

Gene ID	Predicted function [1]	IMG Product Name [2]	Homologs [3]	Predicted localization [4]	Molecular mass (kDa) [5]	Enzymatic and other domains [6]
2842104189	Candidate cellulase	Aryl-phospho-beta-D-glucosidase BglC	*Chitinophaga niabensis* (71% identity)	Unknown	43.3	GH5
2842105209	Candidate cellulase	Endoglucanase	*Chitinophagaceae bacterium* BR5-29 (70% identity)	Unknown, non-Cytoplasmic	40.1	GH5
2842104157	related to cellulase	Endoglucanase	*Cytophagaceae bacterium* SJW1-29 (58% identity)	Unknown, non-Cytoplasmic	65.2	GH9
2842108135	related to chitinase	chitinase	*Chitinophagaceae bacterium* PMP191F (58% identity)	Cytoplasmic	43.2	GH18
2842105133	related to beta-N-acetylhexosaminidase	hypothetical protein	*Chitinophaga* sp. BN140078 (52% identity)	Unknown	98.6	GH20
2842107906	related to beta-N-acetylhexosaminidase	hexosaminidase	*Flavobacteriales bacterium* (50% identity)	Outer membrane (Beta-N-acetylhexosaminidase, Chitobiase)	95.5	GH20-CHB_HEX
2842108966	related to beta-N-acetylhexosaminidase	hexosaminidase	*Cytophagaceae bacterium* (56% identity)	Periplasmic	61.0	GH20
2842106454	related to beta-N-acetylhexosaminidase	hypothetical protein	*Runella* sp. YX9 (63% identity)	Periplasmic	78.9	GH20
2842106655	related to beta-N-acetylhexosaminidase	hexosaminidase	*Cytophagaceae bacterium* SJW1-29 (63% identity)	Outer membrane	97.1	GH20-CHB_HEX
2842109900	related to beta-N-acetylhexosaminidase	hypothetical protein	*Chitinophaga* sp. XS-30 (62% identity)	Periplasmic	84.7	GH20
2842105702	candidate beta-glucanase	beta-glucanase	*Dyadobacter soli* (70% identity)	Extracellular	32.17	GH16
2842104231	Candidate beta-xylosidase	beta-xylosidase	*Cytophagaceae bacterium* SAT51 (78.60% identity)	Unknown, non-Cytoplasmic	36.79	GH43
2842108192	Related to alpha-D-xyloside xylohydrolase	alpha-D-xyloside xylohydrolase	*Chitinophagaceae bacterium* PMP191F (68% identity)	Unknown	81.41	GH31

[1] Predicted functions were assigned by routines used for updating the Carbohydrate Active Enzymes database (http://www.cazy.org/) using the following criteria: typically, 70% or greater amino acid identity with a protein domain with a biochemically determined function at the time of analysis resulted in “candidate” status; 30% to 70% amino acid identity with a protein domain with a known function resulted in “related to” status; and less than 30% amino acid identity with a protein domain with a known function resulted in “distantly related to” status. Because the threshold of similarity that correlates with a change of substrate specificity is variable from one glycoside hydrolase family to another, the criteria were tightened or loosened appropriately for several families. All analyses were conducted domain by domain to avoid problems arising from the modular structure of many of the proteins.
[2] The Integrated Microbial Genomes (IMG) Product Name.
[3] Homologs were identified by a BlastP search with the Swiss-Prot database.
[4] Localization was predicted using the default settings of PSORTb (Gardy et al., 2005). Predicted lipoproteins were identified using LipoP.
[5] Predicted molecular mass of the primary product of translation, including any predicted signal peptide.
[6] CHB_HEX, carbohydrate binding domain represents the N-terminal domain in chitobiases and beta-hexosaminidases. It is composed of a beta sandwich structure that is similar in structure to the cellulose binding domain of cellulase from *Cellulomonas fimi*. (Tews et al., 1996). GH, glycoside hydrolase as assigned by CAZY (the numbers indicate families).

Supplemental TABLE S4: Predicted *Dyadobacter* sp. HH091 polysaccharide lyases[1].

Gene ID	Predicted function [1]	IMG Product Name [2]	Homologs [3]	Predicted localization [4]	Molecular mass (kDa) [5]	Enzymatic and other domains [6]
2842104046	Candidate pectin or pectate lyases	polygalacturonase	Chitinophaga niabensis (71% identity)	Unknown, Cytoplasmic	73105.54	PL, PG
2842105135	Related to pectin or pectate lyases	polygalacturonase	Chitinophagaceae bacterium (63% identity)	Extracellular	55711.69	PL, PG, GH28
2842105517	Related to pectin or pectate lyases	polygalacturonase	*Flavobacterium reichenbachii* (39% identity)	Extracellular	52862.19	PL, PG, GH28
2842107938	Related to pectin or pectate lyases	polygalacturonase	*Bacteroides cellulosilyticus* (42% identity)	Cytoplasmic	44247.94	PL, PG, GH28
2842108446	Candidate pectin or pectate lyases	polygalacturonase	*Cytophagaceae bacterium* SJW1-29 (74% identity)	Extracellular	49580.00	PL, PG, GH28
2842105518	Related to pectin or pectate lyases	polygalacturonase	*Chitinophagaceae bacterium* BR5-29 (38% identity)	Extracellular	55889.57	Pl, PG, GH28
2842109095	Related to pectin or pectate lyases	hypothetical protein	*Pedobacter* sp. OK628 (32% identity)	Extracellular	67179.60	PL, GH28
2842108200	Exo-poly-alpha-D-galacturonosidase	DNA sulfur modification protein DndE	*Cytophagales bacterium* (60% identity)	Extracellular	85717.11	PL, GH28, GDSL_2
2842108975	Related to Pectin lyase-like	hypothetical protein	*Alcanivorax* sp. (42% identity)	Unknown, Non-Cytoplasmic	51861.62	PL

[1] Predicted functions as assigned by routines used for updating the Carbohydrate-Active Enzymes database (http://www.cazy.org/CAZY/) using the criteria indicated in Table 1.
[2] Localization predicted using the default settings of PSORTb (Gardy, J. L., et al. 2005. Bioinformatics 21:617-623).
[3] Predicted molecular mass of primary product of translation, including any predicted signal peptide.
[4] Modular structure is indicated by abbreviations: GH, glycoside hydrolase as assigned by CAZY (the numbers indicate families) (http://www.cazy.org/CAZY/). PL: polysaccharide lyase (number indicates family) as assigned by CAZY. GDSL-like Lipase, (GDSL esterases and lipases are hydrolytic enzymes with multifunctional properties [1]. This new subclass of lipolytic enzymes possesses a distinct GDSL sequence motif different from the GxSxG motif found in many lipases [2]. Members include; Aeromonas hydrophila lipase, Vibrio mimicus arylesterase, Vibrio parahaemolyticus thermolabile haemolysin, rabbit phospholipase (AdRab-B), and Brassica napus anter-specific proline-rich protein.)

Supplemental TABLE S4: Predicted *Dyadobacter* sp. HH091 polysaccharide lyases[1].

Gene ID	Predicted function [1]	IMG Product Name [2]	Homologs [3]	Predicted localization [4]	Molecular mass (kDa) [5]	Enzymatic and other domains [6]
2842104046	Candidate pectin or pectate lyases	polygalacturonase	Chitinophaga niabensis (71% identity)	Unknown, Cytoplasmic	73105.54	PL, PG
2842105135	Related to pectin or pectate lyases	polygalacturonase	Chitinophagaceae bacterium (63% identity)	Extracellular	55711.69	PL, PG, GH28
2842105517	Related to pectin or pectate lyases	polygalacturonase	*Flavobacterium reichenbachii* (39% identity)	Extracellular	52862.19	PL, PG, GH28
2842107938	Related to pectin or pectate lyases	polygalacturonase	*Bacteroides cellulosilyticus* (42% identity)	Cytoplasmic	44247.94	PL, PG, GH28
2842108446	Candidate pectin or pectate lyases	polygalacturonase	*Cytophagaceae bacterium* SJW1-29 (74% identity)	Extracellular	49580.00	PL, PG, GH28
2842105518	Related to pectin or pectate lyases	polygalacturonase	*Chitinophagaceae bacterium* BR5-29 (38% identity)	Extracellular	55889.57	Pl, PG, GH28
2842109095	Related to pectin or pectate lyases	hypothetical protein	*Pedobacter* sp. OK628 (32% identity)	Extracellular	67179.60	PL, GH28
2842108200	Exo-poly-alpha-D-galacturonosidase	DNA sulfur modification protein DndE	*Cytophagales bacterium* (60% identity)	Extracellular	85717.11	PL, GH28, GDSL_2
2842108975	Related to Pectin lyase-like	hypothetical protein	*Alcanivorax* sp. (42% identity)	Unknown, Non-Cytoplasmic	51861.62	PL

[1] Predicted functions as assigned by routines used for updating the Carbohydrate-Active Enzymes database (http://www.cazy.org/CAZY/) using the criteria indicated in Table 1.
[2] Localization predicted using the default settings of PSORTb (Gardy, J. L., et al. 2005. Bioinformatics 21:617-623).
[3] Predicted molecular mass of primary product of translation, including any predicted signal peptide.
[4] Modular structure is indicated by abbreviations: GH, glycoside hydrolase as assigned by CAZY (the numbers indicate families) (http://www.cazy.org/CAZY/). PL: polysaccharide lyase (number indicates family) as assigned by CAZY. GDSL-like Lipase, (GDSL esterases and lipases are hydrolytic enzymes with multifunctional properties [1]. This new subclass of lipolytic enzymes possesses a distinct GDSL sequence motif different from the GxSxG motif found in many lipases [2]. Members include; Aeromonas hydrophila lipase, Vibrio mimicus arylesterase, Vibrio parahaemolyticus thermolabile haemolysin, rabbit phospholipase (AdRab-B), and Brassica napus anter-specific proline-rich protein.)

Supplemental TABLE S5: Predicted *Dyadobacter* sp. HH091 carbohydrate esterases involved in polysaccharide utilization[1].

Gene ID	Predicted function [1]	IMG Product Name [2]	Homologs [3]	Predicted localization [4]	Molecular mass (kDa) [5]
2842104000	Related to polysaccharide deacetylase	peptidoglycan/xylan/chitin deacetylase (PgdA/CDA1 family)	*Flavobacterium álbum* (54% identity)	Cytoplasmic	25806.77
2842105287	Related to polysaccharide deacetylase	peptidoglycan/xylan/chitin deacetylase (PgdA/CDA1 family)	*Flavobacteriales bacterium* (46.88% identity)	Cytoplasmic	28782.40
2842105600	Related to polysaccharide deacetylase	peptidoglycan/xylan/chitin deacetylase (PgdA/CDA1 family)	*Flavobacterium kingsejongi* (66.30% identity)	Unknown (This protein may have multiple localization sites.)	31934.59
2842106253	Candidate polysaccharide deacetylase	peptidoglycan/xylan/chitin deacetylase (PgdA/CDA1 family)	*Cytophagaceae bacterium* SCN 52-12 (61.96% identity)	Unknown	44623.98
2842108758	Related to polysaccharide deacetylase	peptidoglycan/xylan/chitin deacetylase (PgdA/CDA1 family)	*Cytophagaceae bacterium* SJW1-29 (48.85% identity)	Cytoplasmic	32059.73
2842106071	Candidate polysaccharide deacetylase	Hypothetical protein	*Siphonobacter* sp. BAB-5404 (62% identity)	Cytoplasmic	30585.36
2842104846	Candidate acetylxylan esterase	cephalosporin-C deacetylase-like acetyl esterase	*Cytophagaceae bacterium* SJW1-29 (73.49%)	Unknown	48188.31
2842107040	Candidate acetylxylan esterase	hypothetical protein	*Cytophagaceae bacterium* CAR-16 (64.36%)	Cytoplasmic	42412.16

[1] Predicted functions were assigned by routines used for updating the Carbohydrate Active Enzymes database (http://www.cazy.org/) using the following criteria: typically, 70% or greater amino acid identity with a protein domain with a biochemically determined function at the time of analysis resulted in “candidate” status; 30% to 70% amino acid identity with a protein domain with a known function resulted in “related to” status; and less than 30% amino acid identity with a protein domain with a known function resulted in “distantly related to” status. All analyses were conducted domain by domain to avoid problems arising from the modular structure of many of the proteins.
[2] The Integrated Microbial Genomes (IMG) Product Name
[3] Homologs were identified by a BlastP search with the Swiss-Prot database.
[4] Localization was predicted using the default settings of PSORTb (Gardy et al., 2005).
[5] Predicted molecular mass of the primary product of translation, including any predicted signal peptide.

Supplemental TABLE S6: Overall numbers of sequences and contigs generated for the transcriptome datasets.

	Bacteria-exponential phase	Bacteria-stationery phase	Eukaryotic-exponential phase	Eukaryotic-stationery phase
**Reads Illumina (filtered)**				
Total no.	79,956,806	98,243,390	73,776,383	76,211,700
Average length (bp)	73	74	168	177
Duplicates (%)	79.0	75.9	90.1	86.0
Fails (%)	45	36	18	18
GC (%)	51	56	56	57
**Contigs-assembly (CoMW)**				
No.	13,259	22,651	39,255	51,222
Total length (bp)	14,577,971	27,842,014	57,814,484	80,131,706
No. ≥ 1000 bp	4,427	8,553	22,528	30,347
N50 size (bp)	1,211	1,453	1,829	1,979
Largest (bp)	16,877	20,249	11,273	16,587
GC (%)	55.67	57.75	56.95	57.24

Supplemental TABLE S7: Protein family comparison of possible competitive and plant-bacteria interaction pathways across the dominant members of microbiome (*Porphyrobacter, Variovorax* and *Dyadobacter*) of *S. quadricauda* MZCH 10104. Numbers of proteins are marked with the following colors: ■ 0, ■ 1, ■ 2, and ■ 3+.

	Porphyrobacter cryptus DSM 12079	*Porphyrobacter* sp. AAP60	*Porphyrobacter* sp. AAP82	*Porphyrobacter* sp. HL-46	*Dyadobacter alkalitolerans* DSM 23607	*Dyadobacter beijingensis strain* CGMCC 1.6375	*Dyadobacter crusticola* DSM 16708	*Dyadobacter* sp. HH091 *	*Dyadobacter tibetensis* Y620-1	*Variovorax paradoxus* B4	*Variovorax paradoxus* EPS	*Variovorax paradoxus* S110	*Variovorax* sp. 770b2	*Variovorax* sp. CF313	*Variovorax* sp. URHB0020
**Secretion systems**															
**T1SS**															
T1SS membrane fusion protein, HlyD family	0	0	0	0	0	0	0	0	0	1	1	1	1	1	0
T1SS ATPase	0	0	1	0	0	0	0	0	1	1	2	1	0	3+	1
T1SS ATPase, LssB family LapB	0	0	0	0	0	0	0	0	0	1	1	1	1	1	0
**T2SS, T4SS**															
T2SS, T4SS ATP hydrolase TadA/VirB11/CpaF, TadA subfamily	1	1	1	0	0	0	0	0	1	2	2	2	3+	2	0
T2SS, T4SS ATPase TadZ/CpaE, associated with Flp pilus assembly	1	1	1	0	0	0	0	0	1	1	1	0	0	1	0
T2SS, T4SS protein TadC, associated with Flp pilus assembly	1	1	1	0	0	0	0	0	1	1	1	0	0	1	0
T2SS, T4SS protein TadC, associated with Flp pilus assembly	0	0	0	0	0	0	0	0	0	1	1	1	1	1	0
T2SS, T4SS secretin RcpA/CpaC, associated with Flp pilus assembly	1	1	1	0	0	0	0	0	1	2	2	1	1	2	0
T4SS component, VirD4	0	0	0	0	1	1	1	1	1	1	0	1	1	0	0
**T5SS**															
Protein translocase subunit SecA	1	1	1	1	1	1	1	1	1	1	1	1	1	1	0

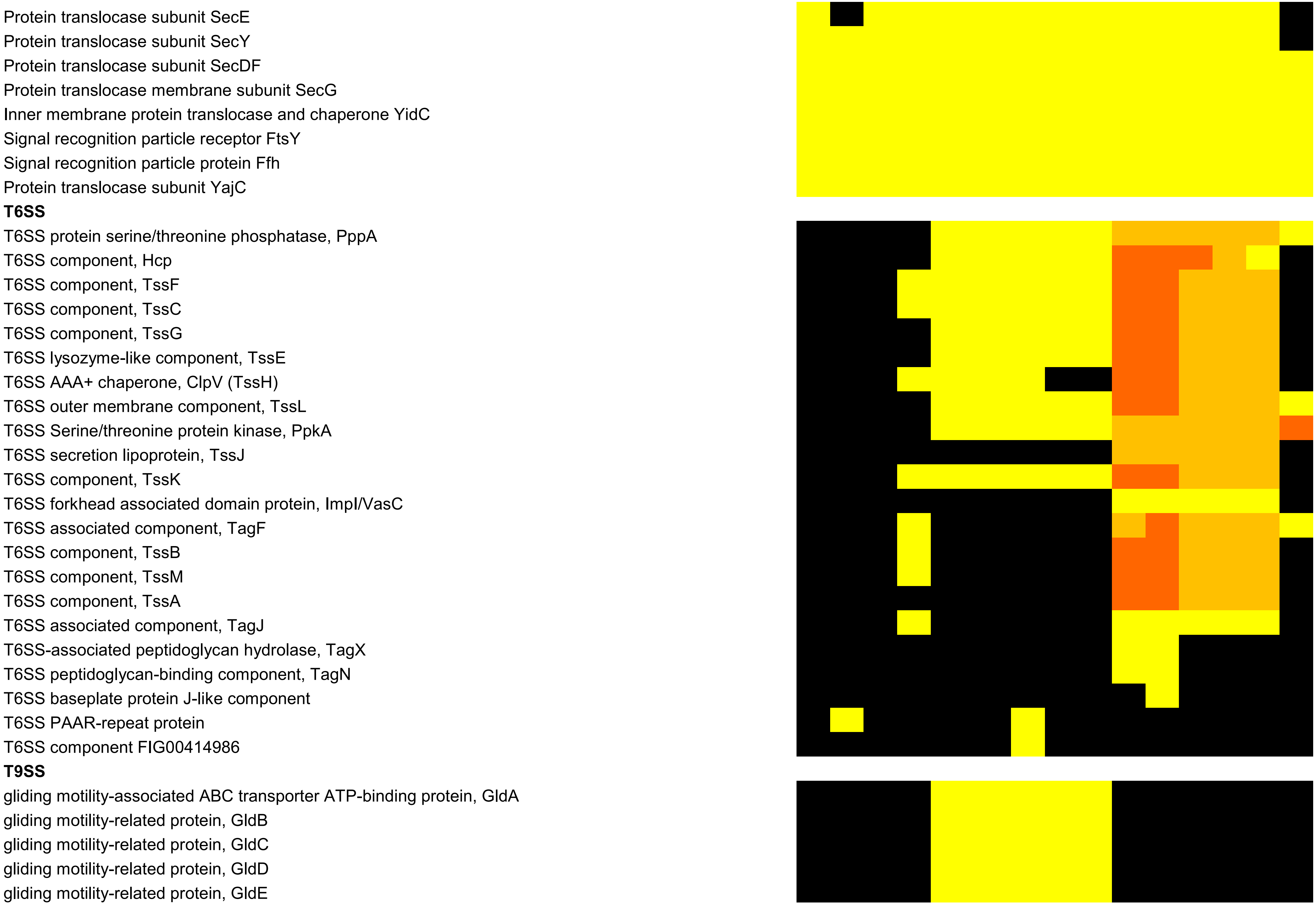
Protein translocase subunit SecE
Protein translocase subunit SecY
Protein translocase subunit SecDF
Protein translocase membrane subunit SecG
Inner membrane protein translocase and chaperone YidC
Signal recognition particle receptor FtsY
Signal recognition particle protein Ffh
Protein translocase subunit YajC
T6SS
T6SS protein serine/threonine phosphatase, PppA
T6SS component, Hcp
T6SS component, TssF
T6SS component, TssC
T6SS component, TssG
T6SS lysozyme-like component, TssE
T6SS AAA+ chaperone, ClpV (TssH)
T6SS outer membrane component, TssL
T6SS Serine/threonine protein kinase, PpkA
T6SS secretion lipoprotein, TssJ
T6SS component, TssK
T6SS forkhead associated domain protein, ImpI/VasC
T6SS associated component, TagF
T6SS component, TssB
T6SS component, TssM
T6SS component, TssA
T6SS associated component, TagJ
T6SS-associated peptidoglycan hydrolase, TagX
T6SS peptidoglycan-binding component, TagN
T6SS baseplate protein J-like component
T6SS PAAR-repeat protein
T6SS component FIG00414986
T9SS
gliding motility-associated ABC transporter ATP-binding protein, GldA
gliding motility-related protein, GldB
gliding motility-related protein, GldC
gliding motility-related protein, GldD
gliding motility-related protein, GldE

gliding motility-associated ABC transporter permease protein, GldF
gliding motility-associated ABC transporter substrate-binding protein, GldG
gliding motility-related protein, GldH
gliding motility-related protein, GldL
gliding motility-related protein, GldM
gliding motility-related protein, GldN and/or GldO
gliding motility-related protein, SprA
gliding motility-related protein, SprB
gliding motility-related protein, SprE
gliding motility-related protein, SprF

Response and transcriptional regulators

Two-component transcriptional response regulator, LuxR family
Acyl-homoserine lactone-binding transcriptional activator, LuxR family
Two-component transcriptional response regulator, LuxR family, but with unusual receiver domain
Transcriptional regulator, LuxR family
Two-component transcriptional response regulator, NarL/FixJ family
RNA polymerase sigma-70 factor
Sigma-70 factor

Polysaccharides degradation/modification

GH

beta-galactosidase, GH2, GH1, GH35, GH39, GH42 (EC 3.2.1.23)
alpha-L-arabinofuranosidase, GH2, GH39, GH62 (EC 3.2.1.55)
beta-mannosidase, GH2 (EC 3.2.1.25)
beta-glucosidase, GH2, GH1, GH30, GH39 (EC 3.2.1.21)
beta-glycosyl hydrolase, GH3
mannan endo-1,4-beta-mannosidase, GH5, GH26 (EC:3.2.1.78)
beta-1,4-glucanase (cellulase), GH9, GH6, GH7, GH8, GH45 (EC 3.2.1.4)
endo-1,4-beta-xylanase, GH10, GH8, GH30 (EC 3.2.1.8)
xylanase, GH11 (EC:3.2.1.-)
alpha-amylase, GH13 (EC 3.2.1.1)
malto-oligosyltrehalose synthase , GH13 (EC 5.4.99.15)
Chitinase, GH18, GH19 (EC 3.2.1.14)
N-acetyl-beta-hexosaminidase, GH20, GH84 (EC 3.2.1.52)
beta-N-acetylglucosaminidase, GH20 (EC 3.2.1.52)

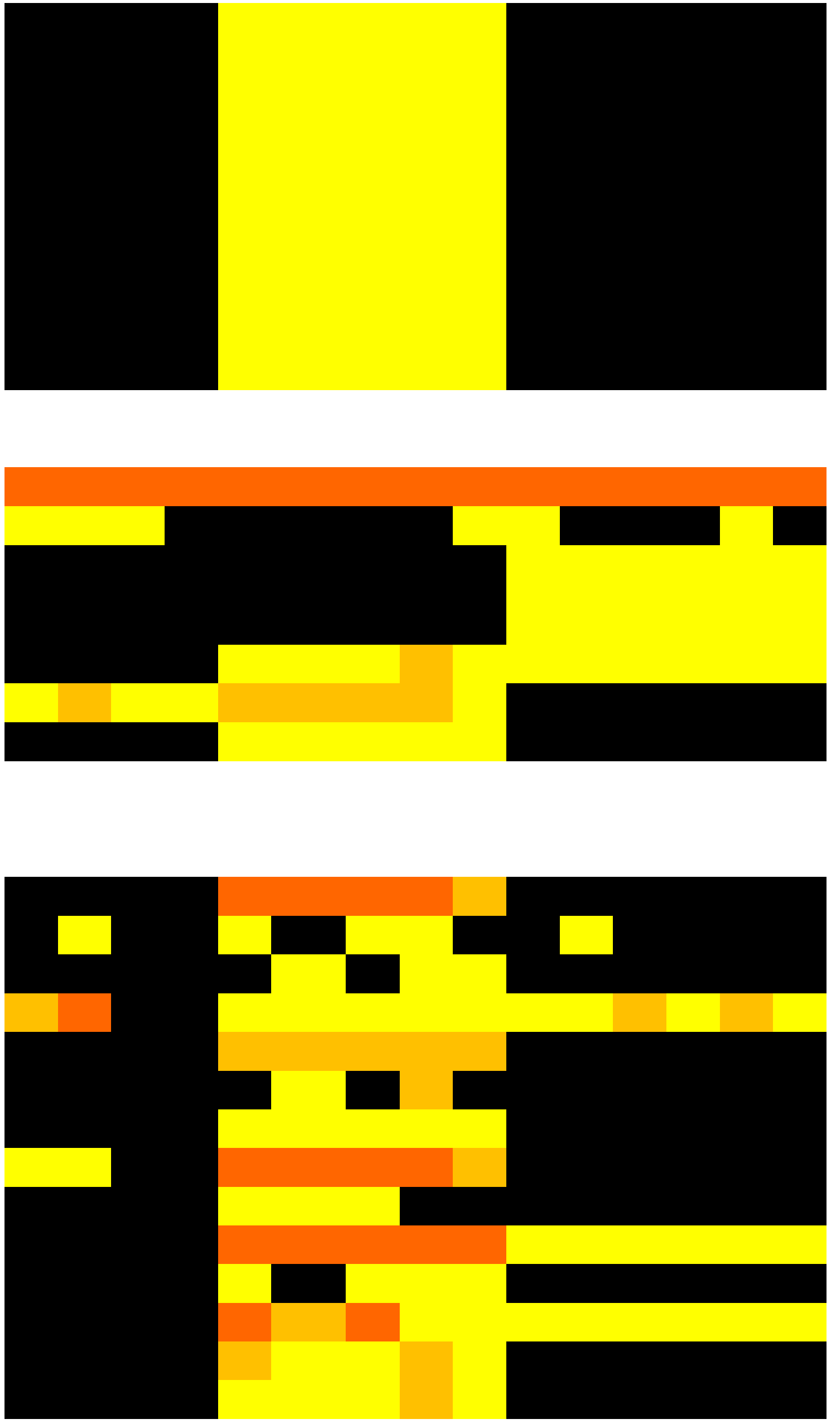

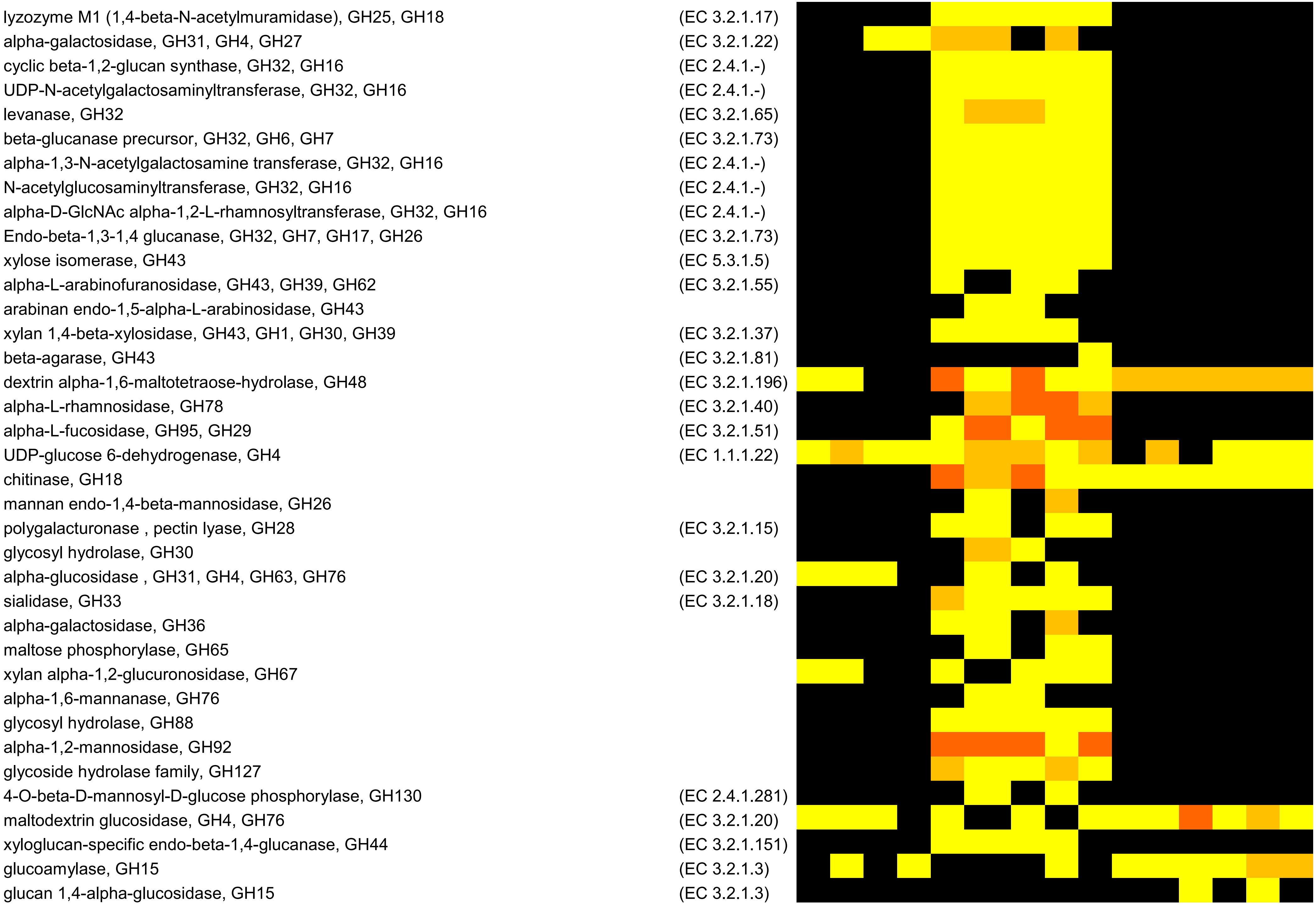
lyzozyme M1 (1,4-beta-N-acetylmuramidase), GH25, GH18 (EC 3.2.1.17)
alpha-galactosidase, GH31, GH4, GH27 (EC 3.2.1.22)
cyclic beta-1,2-glucan synthase, GH32, GH16 (EC 2.4.1.-)
UDP-N-acetylgalactosaminyltransferase, GH32, GH16 (EC 2.4.1.-)
levanase, GH32 (EC 3.2.1.65)
beta-glucanase precursor, GH32, GH6, GH7 (EC 3.2.1.73)
alpha-1,3-N-acetylgalactosamine transferase, GH32, GH16 (EC 2.4.1.-)
N-acetylglucosaminyltransferase, GH32, GH16 (EC 2.4.1.-)
alpha-D-GlcNAc alpha-1,2-L-rhamnosyltransferase, GH32, GH16 (EC 2.4.1.-)
Endo-beta-1,3-1,4 glucanase, GH32, GH7, GH17, GH26 (EC 3.2.1.73)
xylose isomerase, GH43 (EC 5.3.1.5)
alpha-L-arabinofuranosidase, GH43, GH39, GH62 (EC 3.2.1.55)
arabinan endo-1,5-alpha-L-arabinosidase, GH43
xylan 1,4-beta-xylosidase, GH43, GH1, GH30, GH39 (EC 3.2.1.37)
beta-agarase, GH43 (EC 3.2.1.81)
dextrin alpha-1,6-maltotetraose-hydrolase, GH48 (EC 3.2.1.196)
alpha-L-rhamnosidase, GH78 (EC 3.2.1.40)
alpha-L-fucosidase, GH95, GH29 (EC 3.2.1.51)
UDP-glucose 6-dehydrogenase, GH4 (EC 1.1.1.22)
chitinase, GH18
mannan endo-1,4-beta-mannosidase, GH26
polygalacturonase , pectin lyase, GH28 (EC 3.2.1.15)
glycosyl hydrolase, GH30
alpha-glucosidase , GH31, GH4, GH63, GH76 (EC 3.2.1.20)
sialidase, GH33 (EC 3.2.1.18)
alpha-galactosidase, GH36
maltose phosphorylase, GH65
xylan alpha-1,2-glucuronosidase, GH67
alpha-1,6-mannanase, GH76
glycosyl hydrolase, GH88
alpha-1,2-mannosidase, GH92
glycoside hydrolase family, GH127
4-O-beta-D-mannosyl-D-glucose phosphorylase, GH130 (EC 2.4.1.281)
maltodextrin glucosidase, GH4, GH76 (EC 3.2.1.20)
xyloglucan-specific endo-beta-1,4-glucanase, GH44 (EC 3.2.1.151)
glucoamylase, GH15 (EC 3.2.1.3)
glucan 1,4-alpha-glucosidase, GH15 (EC 3.2.1.3)

trehalase, GH15, GH37	(EC 3.2.1.28)
beta-porphyranase, GH16	(EC 3.2.1.178)
alpha-galactosidase, GH31	(EC 3.2.1.22)
alpha-xylosidase, GH31	(EC 3.2.1.177)
1,4-alpha-glucan branching enzyme, GH13	(EC 2.4.1.18)
4-alpha-glucanotransferase (amylomaltase), GH57, GH77, GH13	(EC 2.4.1.25)
xylan alpha-1,2-glucuronosidase, GH67	(EC 3.2.1.131)
alpha-N-acetylglucosaminidase, GH89	(EC 3.2.1.50)
Maltose phosphorylase, GH65	(EC 2.4.1.8)
4-O-beta-D-mannosyl-D-glucose phosphorylase, GH130	(EC 2.4.1.281)

GT

glycosyltransferase, GT1	
N-acetylglucosaminyltransferase, GT1, GT2, GT4, GT5	(EC 2.4.1.-)
b-glycosyltransferase, GT2	
glycosyltransferase YkoT, GT2	
a-glycosyltransferase-related protein, GT4	
alpha-1,3-N-acetylgalactosamine transferase PglA, GT4, GT5	(EC 2.4.1.-)
alpha-1,2-fucosyltransferase, GT11	
trehalose-6-phosphate phosphatase, GT20	(EC 3.1.3.15)
UDP-N-acetylglucosamine:L-malate glycosyltransferase, GT41	
alpha-D-GlcNAc alpha-1,2-L-rhamnosyltransferase, GT1, GT2, GT4, GT5	
lipid carrier : UDP-N-acetylgalactosaminyltransferase, GT1, GT2, GT5	(EC 2.4.1.-)
cyclic beta-1,2-glucan synthase, GT2, GT4, GT5	(EC 2.4.1.-)
Glycogen phosphorylase, GT35	(EC 2.4.1.1)
Penicillin-insensitive transglycosylase, GT4	(EC 2.4.2.-)
Similar to glycogen synthase, GT5	(EC 2.4.1.21)
Glycogen synthase, ADP-glucose transglucosylase, GT5	(EC 2.4.1.21)
Lipid-A-disaccharide synthase, GT19	(EC 2.4.1.182)
Similar to glycogen synthase, GT3	(EC 2.4.1.21)
UDP-N-acetylglucosamine--N-acetylmuramyl-(pentapeptide) pyrophosphoryl-undecaprenol N-acetylglucosamine transferase, GT28	(EC 2.4.1.227)
Biofilm PGA synthesis N-glycosyltransferase PgaC, GT2	(EC 2.4.-.-)
1,2-diacylglycerol 3-glucosyltransferase, GT28	(EC 2.4.1.157)
Cellulose synthase, GT2	(EC 2.4.1.12)
Cyclic beta-1,2-glucan synthase, GT84	(EC 2.4.1.-)
Dolichol-phosphate mannosyltransferase, GT2	(EC 2.4.1.83)

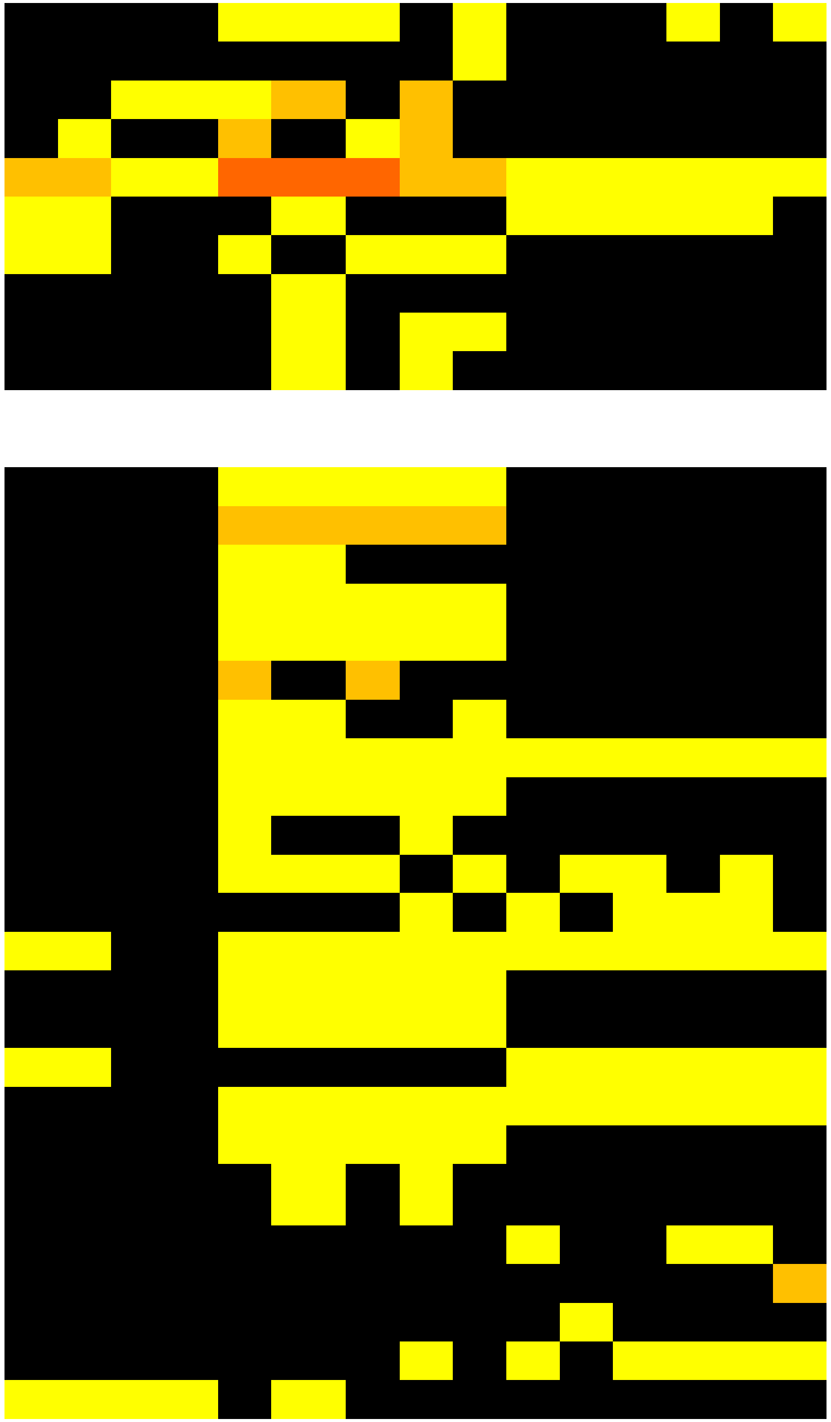

Multimodular transpeptidase-transglycosylase, GT51
Membrane carboxypeptidase (penicillin-binding protein), GT51

CE
carbohydrate esterase, CE1
indoleacetamide hydrolase, CE4 (EC 3.5.1.-)
polysaccharide deacetylase, CE4

PL
alginate lyase precursor, PL7 (EC 4.2.2.3)
Pectate lyase, PL9 (EC 4.2.2.2)

Peptidases
Peptidase M20
Peptidase S9, alanyl dipeptidyl peptidase
Peptidase M19, Zn-dependent dipeptidase
Peptidase M28, leucine aminopeptidase-related protein
Peptidase M13
Peptidase M14, zinc-dependent carboxypeptidase
Peptidase M16
Peptidase B M17
Peptidase S24
Peptidase M24, Xaa-Pro dipeptidase
Peptidase C82, L,D-transpeptidase
Pepdidase M14, zinc carboxypeptidase domain protein
Peptidase S10, carboxypeptidase-related protein
Peptidase M23/M37
Peptidase S9, dipeptidyl anminopeptidase
Peptidase S9, prolyl oligopeptidase family protein
Peptidase M28, aminopeptidase CC_2544
Peptidase M16, zinc protease
Peptidase S26, signal peptidase I
Peptidase S9, dipeptidyl peptidase IV
Peptidase S49, signal peptide peptidase SppA (protease 4)
Peptidase M48
Peptidase M23, membrane proteins related to metalloendopeptidases
Peptidase T2, isoaspartyl aminopeptidase

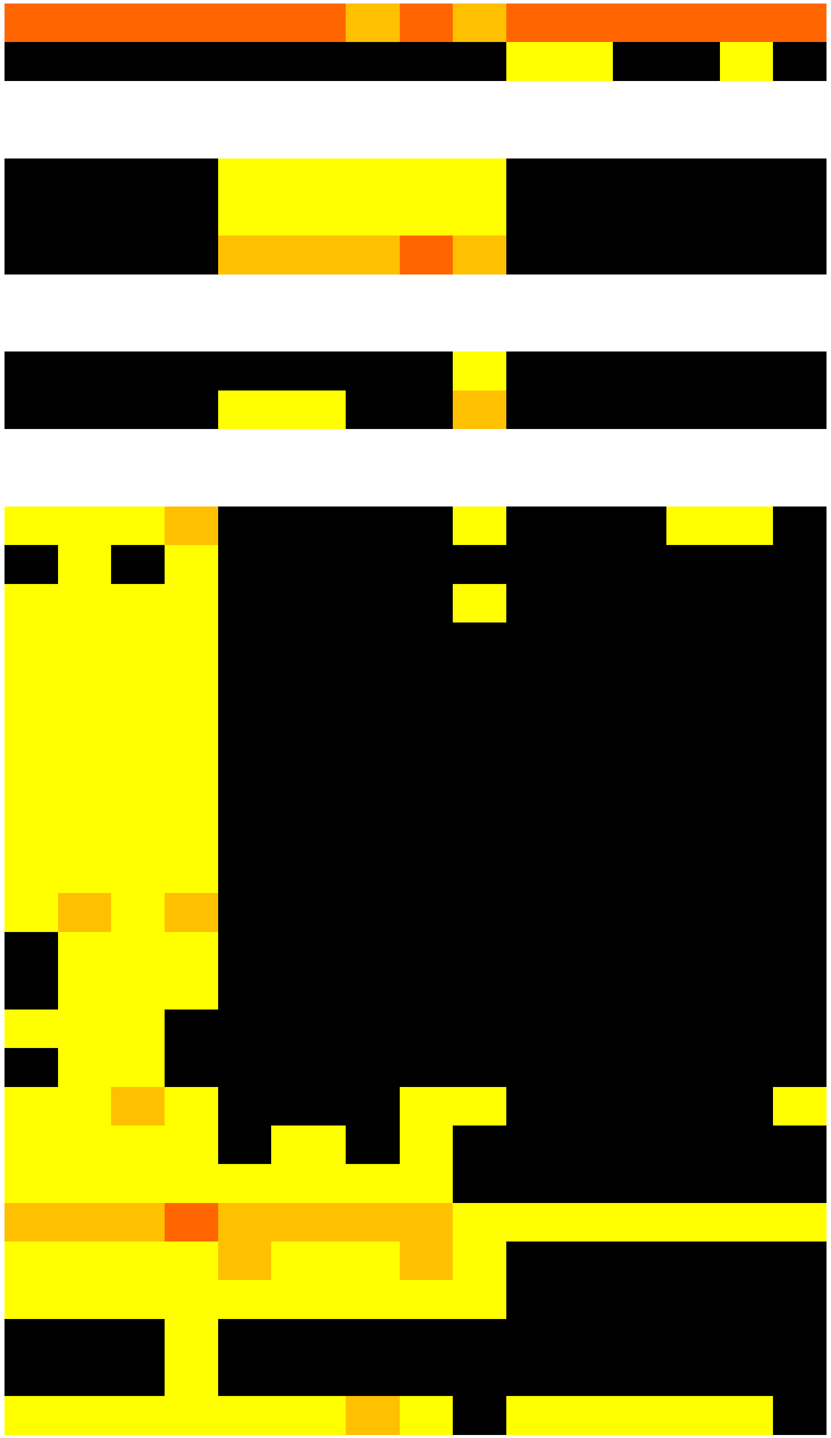

Peptidase M13, metallopeptidase
Peptidase M17, cytosol aminopeptidase PepA
Peptidase S9, prolyl endopeptidase
Peptidase M1, Zn-dependent aminopeptidase
Peptidase M14, carboxypeptidase A precursor
Peptidase M23/M37
Peptidase M28, aminopeptidase Y
Peptidase M49, dipeptidyl-peptidase III
Peptidase M10A/M12B, matrixin/adamalysin
Peptidase C39
Peptidase M28, glutamate carboxypeptidase II
Peptidase M61
Peptidase M28, aminopeptidase
Peptidase M1, aminopeptidase
Peptidase M28, putative aminopeptidase
Peptidase M24B, Xaa-Pro aminopeptidase
Peptidase M28, aminopeptidase
Peptidase M23B
Peptidase M1, putative aminopeptidase
Peptidase M13
Peptidase S41, C-terminal processing peptidase, tail-specific protease
Peptidase M28
peptidase M19, renal dipeptidase
Peptidase M1
Peptidase M15, D-alanyl-D-alanine dipeptidase
Peptidase M20B, tripeptide aminopeptidase
Peptidase M16
Peptidase Y M28, aminopeptidase Y
Peptidase M1, membrane alanine aminopeptidase N
Peptidase M3, oligoendopeptidase F-like protein
Peptidase M28, putative aminopeptidase
Peptidase A8, lipoprotein signal peptidase
Peptidase M16
Peptidase S9, X-Pro dipeptidyl-peptidase
Peptidase S8/S53, subtilisin/kexin/sedolisin
Peptidase M14, N-terminal Zinc-dependent carboxypeptidase
Peptidase M1, aminopeptidase N-like protein

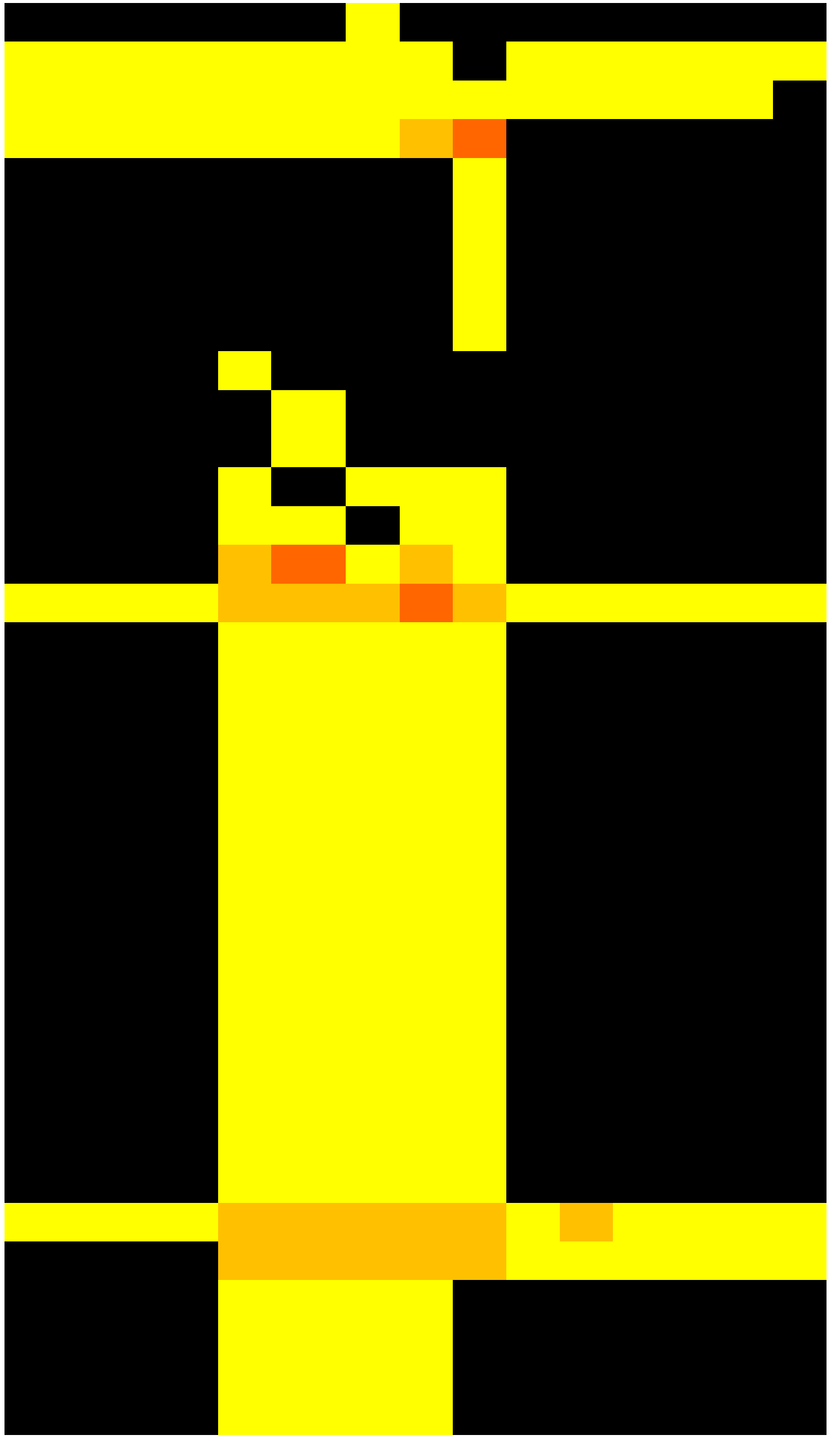

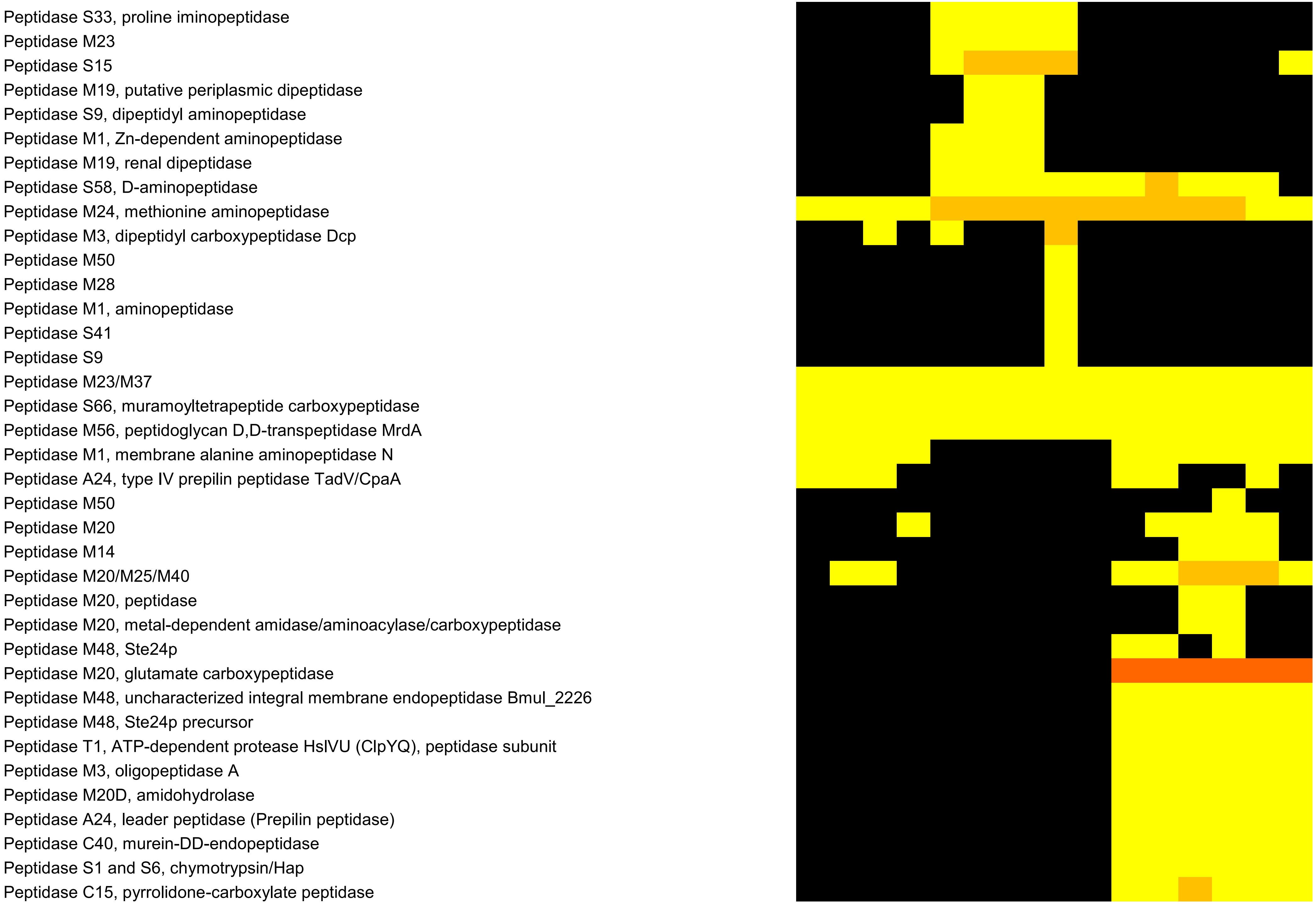
Peptidase S33, proline iminopeptidase
Peptidase M23
Peptidase S15
Peptidase M19, putative periplasmic dipeptidase
Peptidase S9, dipeptidyl aminopeptidase
Peptidase M1, Zn-dependent aminopeptidase
Peptidase M19, renal dipeptidase
Peptidase S58, D-aminopeptidase
Peptidase M24, methionine aminopeptidase
Peptidase M3, dipeptidyl carboxypeptidase Dcp
Peptidase M50
Peptidase M28
Peptidase M1, aminopeptidase
Peptidase S41
Peptidase S9
Peptidase M23/M37
Peptidase S66, muramoyltetrapeptide carboxypeptidase
Peptidase M56, peptidoglycan D,D-transpeptidase MrdA
Peptidase M1, membrane alanine aminopeptidase N
Peptidase A24, type IV prepilin peptidase TadV/CpaA
Peptidase M50
Peptidase M20
Peptidase M14
Peptidase M20/M25/M40
Peptidase M20, peptidase
Peptidase M20, metal-dependent amidase/aminoacylase/carboxypeptidase
Peptidase M48, Ste24p
Peptidase M20, glutamate carboxypeptidase
Peptidase M48, uncharacterized integral membrane endopeptidase Bmul_2226
Peptidase M48, Ste24p precursor
Peptidase T1, ATP-dependent protease HslVU (ClpYQ), peptidase subunit
Peptidase M3, oligopeptidase A
Peptidase M20D, amidohydrolase
Peptidase A24, leader peptidase (Prepilin peptidase)
Peptidase C40, murein-DD-endopeptidase
Peptidase S1 and S6, chymotrypsin/Hap
Peptidase C15, pyrrolidone-carboxylate peptidase

Peptidase M23, membrane proteins related to metalloendopeptidases
Peptidase M48, Ste24p
Peptidase M55, D-aminopeptidase dipeptide-binding protein DppA
Peptidase C14, caspase catalytic subunit p20
Peptidase C82, L,D-transpeptidase
Peptidase T3, gamma-glutamyltranspeptidase
Peptidase S33, proline iminopeptidase
Peptidase S01A, lysyl endopeptidase
Peptidase M24
Peptidase C39, bacteriocin resistance protein
Peptidase S33, proline iminopeptidase
Peptidase C39, ABC-type bacteriocin/lantibiotic exporters, contain an N-terminal double-glycine peptidase domain
Peptidase S11, D-alanyl-D-alanine carboxypeptidase

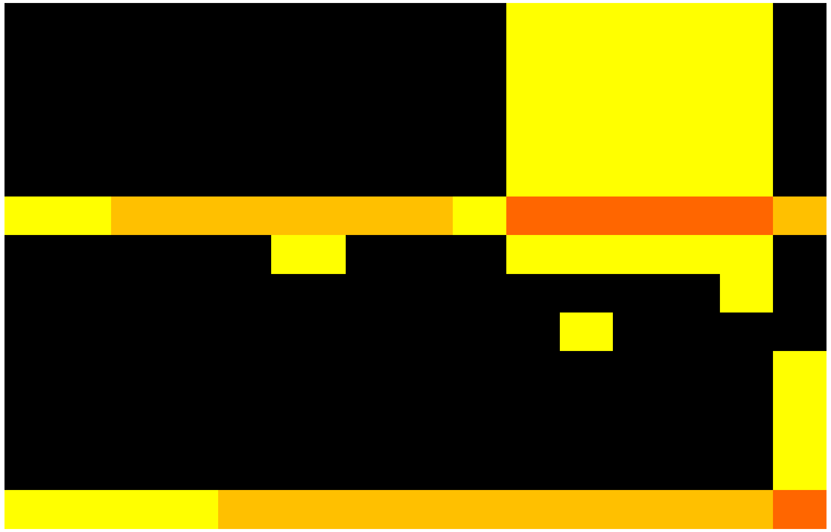

Endonucleases and exonucleases

Single-stranded-DNA-specific exonuclease RecJ
Endonuclease III
HNH endonuclease
mRNA 3-end processing exonuclease
endonuclease
probable DNA repair exonuclease
Ribonuclease J (endonuclease and 5' exonuclease)
Type II restriction endonuclease
Type III restriction system endonuclease
Endonuclease/exonuclease/phosphatase
Endonuclease V
Exonuclease SbcC
Exonuclease SbcD
IncQ plasmid conjugative transfer DNA nicking endonuclease TraR
3'-5' exonuclease
Endonuclease containing a URI domain
DNA repair exonuclease family protein YhaO
CRISPR-associated RecB family exonuclease Cas4
tRNA 3 endonuclease
CRISPR-associated endonuclease Cas9
Endonuclease NmeDIP

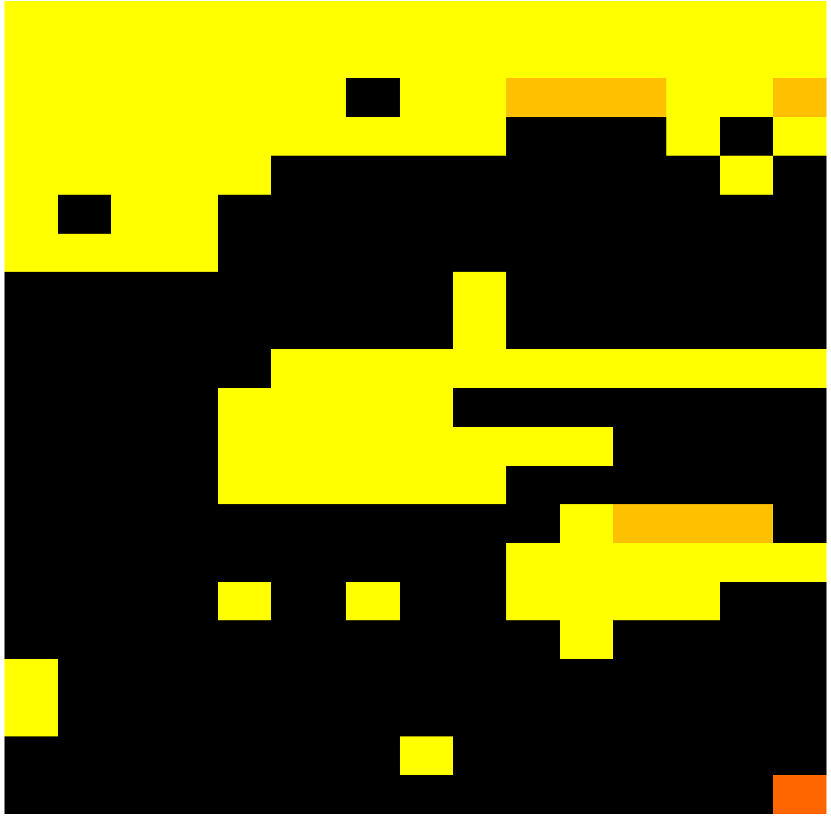

Heme synthesis

Uroporphyrinogen-III synthase, HemD
Coproporphyrinogen III oxidase, aerobic (EC 1.3.3.3), CPOX, HemF
Coproporphyrinogen III oxidase, oxygen-independent
Porphobilinogen synthase, HemB
Glutamyl-tRNA synthetase,GltX
Protoporphyrinogen IX oxidase, novel form, HemJ
Ferrochelatase, protoheme ferro-lyase, HemH
Heme A synthase, cytochrome oxidase biogenesis protein, Cox15-CtaA
Glutamate-1-semialdehyde 2,1-aminomutase, HemL
Glutamyl-tRNA reductase, HemA

QQ

6-phosphogluconolactonase
dienelactone hydrolase
gluconolactonase
metal-dependent hydrolase
NUDIX hydrolase
acyl-homoserine lactone acylase PvdQ (EC 3.5.1.-), quorum-quenching
Zn-dependent hydrolase of the beta-lactamase fold-like protein

QS

N-acyl-L-homoserine lactone synthase, LuxI family (EC 2.3.1.184)
Dialkylrecorsinol condensing enzyme, DarA
3-oxoacyl-[acyl-carrier-protein] synthase III, DarB (EC 2.3.1.41)

Vitamins biosynthesis

Thiamine biosynthesis, B1

2-iminoacetate synthase, ThiH
Hydroxyethylthiazole kinase
Thiamin pyrophosphokinase
Thiamine monophosphate synthase
Thiamine-monophosphate kinase
Thiazole synthase, ThiGH
Phosphomethylpyrimidine synthase, ThiC
Sulfur carrier protein, ThiS

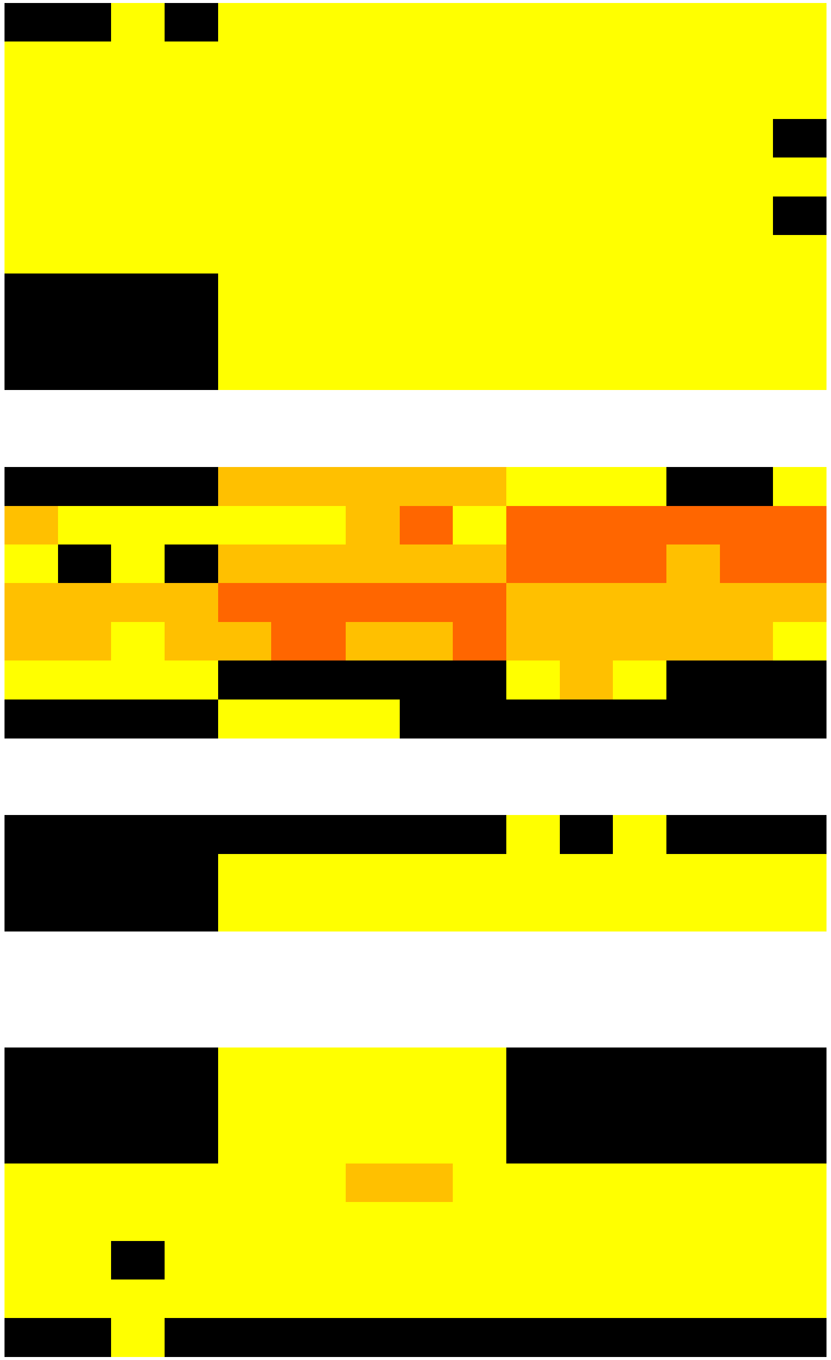

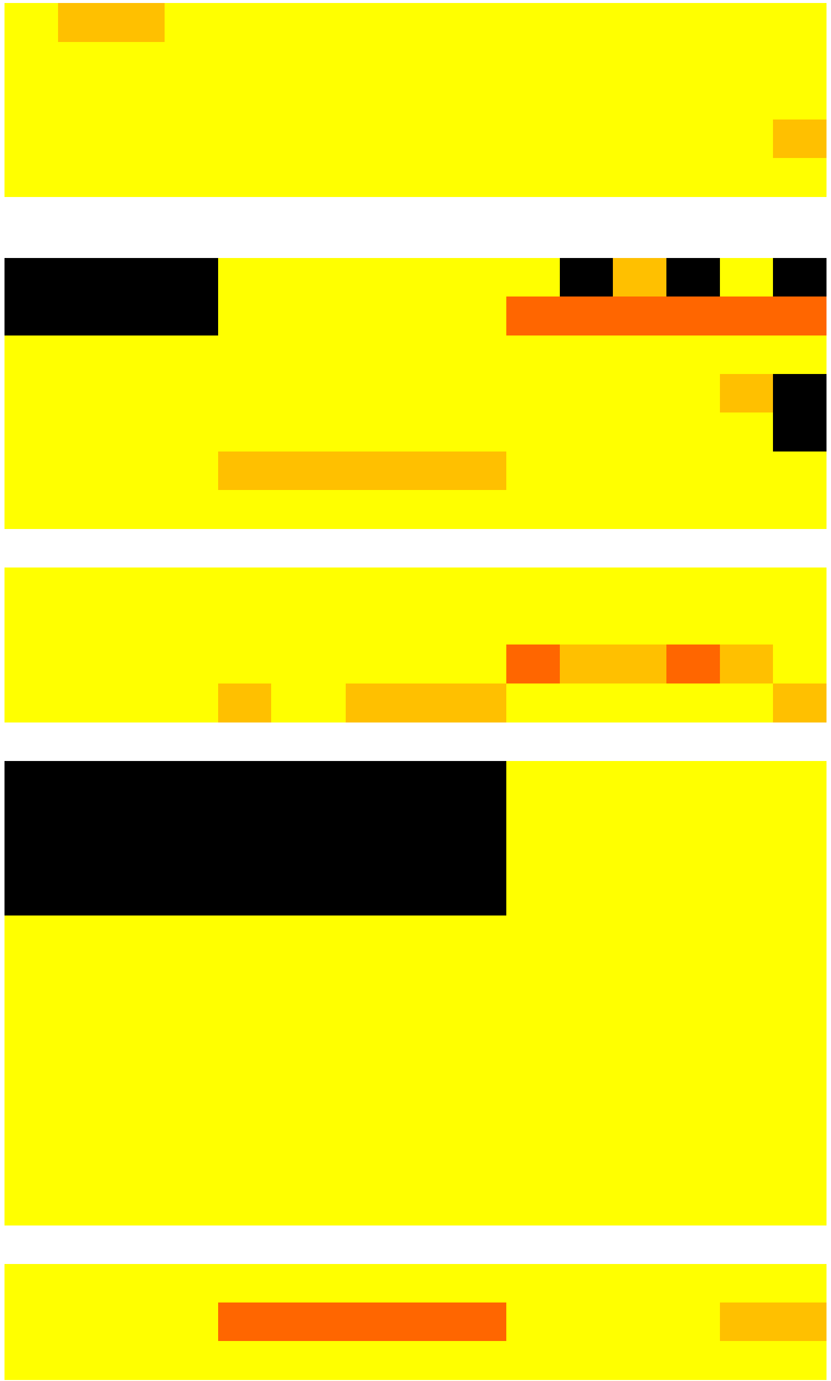

Riboflavin/FAD biosynthesis, B2

GTP cyclohydrolase II

6,7-dimethyl-8-ribityllumazine synthase, riboflavin synthase beta chain

Pyrimidine reductase, riboflavin biosynthesis

FMN adenylyltransferase / Riboflavin kinase

Riboflavin synthase eubacterial/eukaryotic

Pantothenate/CoA biosynthesis, B5

Aspartate 1-decarboxylase

Ketopantoate reductase

Dephospho-CoA kinase

Ketopantoate hydroxymethyltransferase

Panthothenate synthetase

Pantothenate kinase type III, CoaX-like

Phosphopantetheine adenylyltransferase

Pyridoxal 5'-phosphate, B6

Pyridoxamine 5'-phosphate oxidase (EC 1.4.3.5)

Pyridoxine 5'-phosphate synthase (EC 2.6.99.2)

4-hydroxythreonine-4-phosphate dehydrogenase (EC 1.1.1.262)

Phosphoserine aminotransferase (EC 2.6.1.52)

Biotin biosynthesis, B7

Adenosylmethionine-8-amino-7-oxononanoate aminotransferase, BioA

Dethiobiotin synthetase, BioD

7-keto-8-aminopelargonate synthetase or related enzyme, BioF

malonyl-CoA O-methyltransferase, BioC

3-oxoacyl-[acyl-carrier-protein] synthase I, FabB

3-oxoacyl-[acyl-carrier-protein] synthase II, FabF

3-oxoacyl-[acyl-carrier protein] reductase, FabG

beta-hydroxyacyl-(acyl-carrier-protein) dehydratase, FabZ

enoyl-[acyl-carrier protein] reductase I, FabI

Biotin synthase, BioB

Biotin-protein ligase. BirA2

8-amino-7-oxononanoate synthase

Folate biosynthesis, B9

2-amino-4-hydroxy-6-hydroxymethyldihydropteridine pyrophosphokinase

Dihydrofolate reductase

Dihydroneopterin aldolase

Dihydropteridine reductase
FMN reductase (NADPH)

Cobalamine/B12 biosynthesis

Cobalamin synthase, CobS (EC 2.7.8.26)
alpha ribazole-5'-P phosphatase in cobalamin synthesis, CobC
nicotinate-nucleotide dimethylbenzimidazole-P phophoribosyl transferase, CobT
adenosylcobinamide kinase/adenosylcobinamide-phosphate guanylyltransferase, CobU
synthesis of vitamin B12 adenosyl cobalamide precursor, CobD
cob(I)alamin and cobinamide adenolsyltransferase, BtuR, CobA, CobO
Aerobic cobaltochelatase, CobN (EC 6.6.1.2)
cobyrinic acid a,c-diamide synthase, CbiA, CobB (EC 6.3.5.9,11)
synthesis of vitamin B12 adenosyl cobalamide precursor, CbiP
cobyrinic acid a,c-diamide synthase, CobB
precorrin-2 dehydrogenase / sirohydrochlorin ferrochelatase, CysG
Aerobic cobaltochelatase, CobS (EC 6.6.1.2)
Aerobic cobaltochelatase, CobT (EC 6.6.1.2)
sirohydrochlorin cobaltochelatase, CbiK
precorrin-8X/cobalt-precorrin-8 methylmutase, CbiC, CobH
cobalt-precorrin-7 (C5)-methyltransferase, CbiE
cobalt-precorrin-6B (C15)-methyltransferase, CbiT, CobL
cobalt-precorrin-5B (C1)-methyltransferase, CbiD
cobalt-precorrin 5A hydrolase, CbiG
precorrin-4/cobalt-precorrin-4 C11-methyltransferase, CbiF
cobalt-precorrin-3 C(17)-methyltransferase, CbiH
precorrin-2/cobalt-factor-2 C20-methyltransferase, CbiL

Histidine biosynthesis

Hydrolase, HAD superfamily
ATP phosphoribosyltransferase , HisGs
ATP phosphoribosyltransferase regulatory subunit, HisG
Phosphoribosyl-ATP pyrophosphatase, HisI2
ATP phosphoribosyltransferase, HisGl
Phosphoribosyl-AMP cyclohydrolase, HisI1
Phosphoribosylformimino-5-aminoimidazole carboxamide ribotide isomerase, HisA
Ribose-phosphate pyrophosphokinase, PrsA
Imidazole glycerol phosphate synthase amidotransferase, HisH

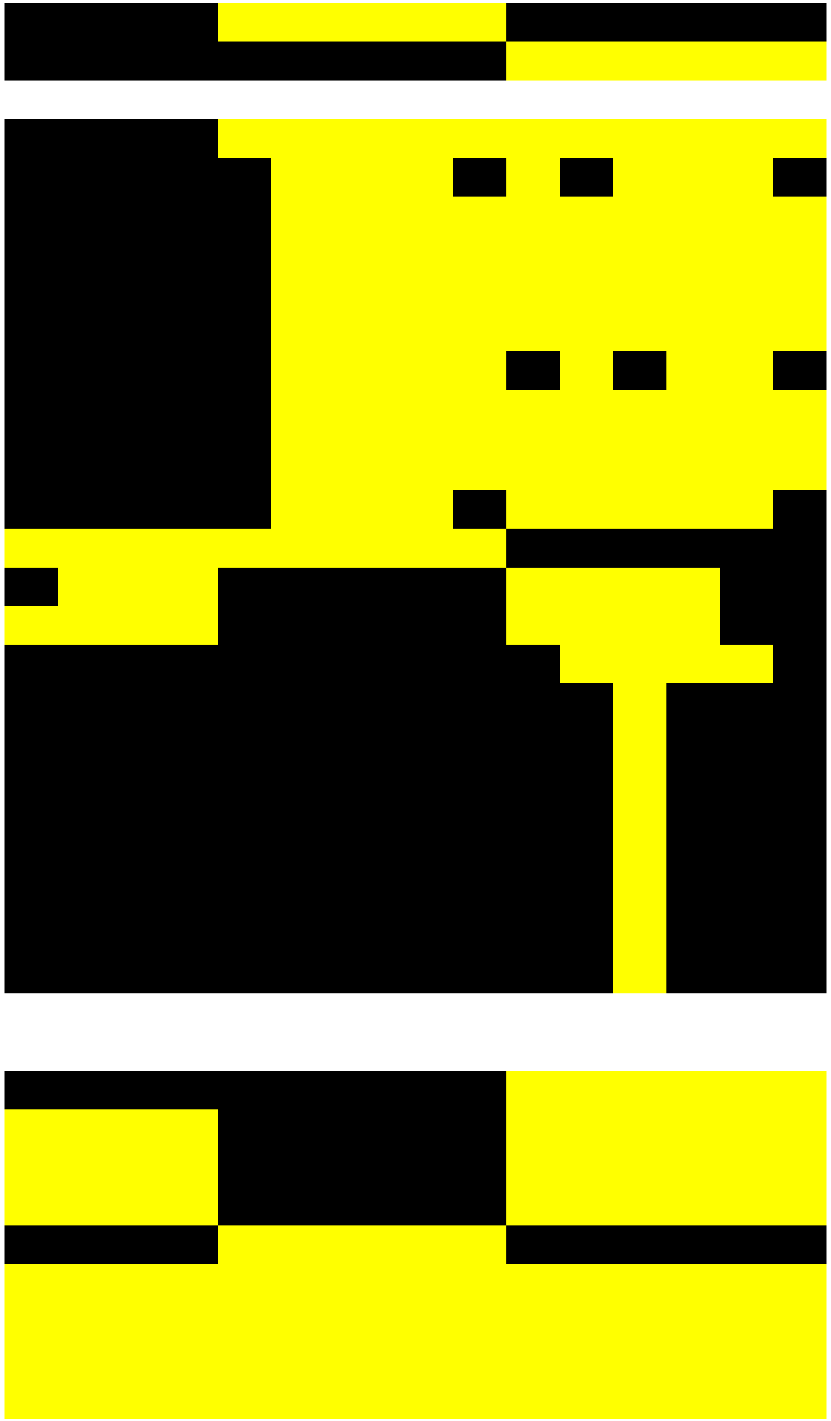

Histidinol dehydrogenase, HisD
Histidinol-phosphatase, HisB1

Bacteriochlorophyll synthesis and phototropic activity

2-desacetyl-2-hydroxyethyl bacteriochlorophyllide A dehydrogenase, BchC
2-vinyl bacteriochlorophyllide hydratase, BchF
Bacteriochlorophyll synthase
Protein BchJ, involved in reduction of C-8 vinyl of divinyl protochlorophyllide
Chlorophyllide reductase subunit, BchX
Chlorophyllide reductase subunit, BchY
Chlorophyllide reductase subunit, BchZ
Light-harvesting LHI, alpha subunit
Photosynthetic reaction center H subunit
Photosynthetic reaction center L subunit
Photosynthetic reaction center M subunit
Photosynthetic complex assembly protein RPC_1320
Protein BchJ, involved in reduction of C-8 vinyl of divinyl protochlorophyllide
Light-independent protochlorophyllide reductase iron-sulfur ATP-binding protein, ChlL
Light-independent protochlorophyllide reductase subunit B
Light-independent protochlorophyllide reductase subunit N
Divinyl protochlorophyllide a 8-vinyl-reductase
Mg-protoporphyrin IX monomethyl ester oxidative cyclase (anaerobic)
Mg protoporphyrin IX monomethyl ester oxidative cyclase (aerobic)

Phenylalanine, tyrosine and tryptophane biosynthesis

2-keto-3-deoxy-D-arabino-heptulosonate-7-phosphate synthase I alpha, AroGA
2-keto-3-deoxy-D-arabino-heptulosonate-7-phosphate synthase I beta, AroG1
3-dehydroquinate synthase, AroB
3-dehydroquinate dehydratase, AroD
Shikimate 5-dehydrogenase I alpha, AroE
Shikimate kinase I, AroK
3-phosphoshikimate 1-carboxyvinyltransferase, AroA
Chorismate synthase, AroC
Chorismate mutase, PheA
Prephenate dehydratase, PheA2
Cyclohexadienyl dehydrogenase, TyrC

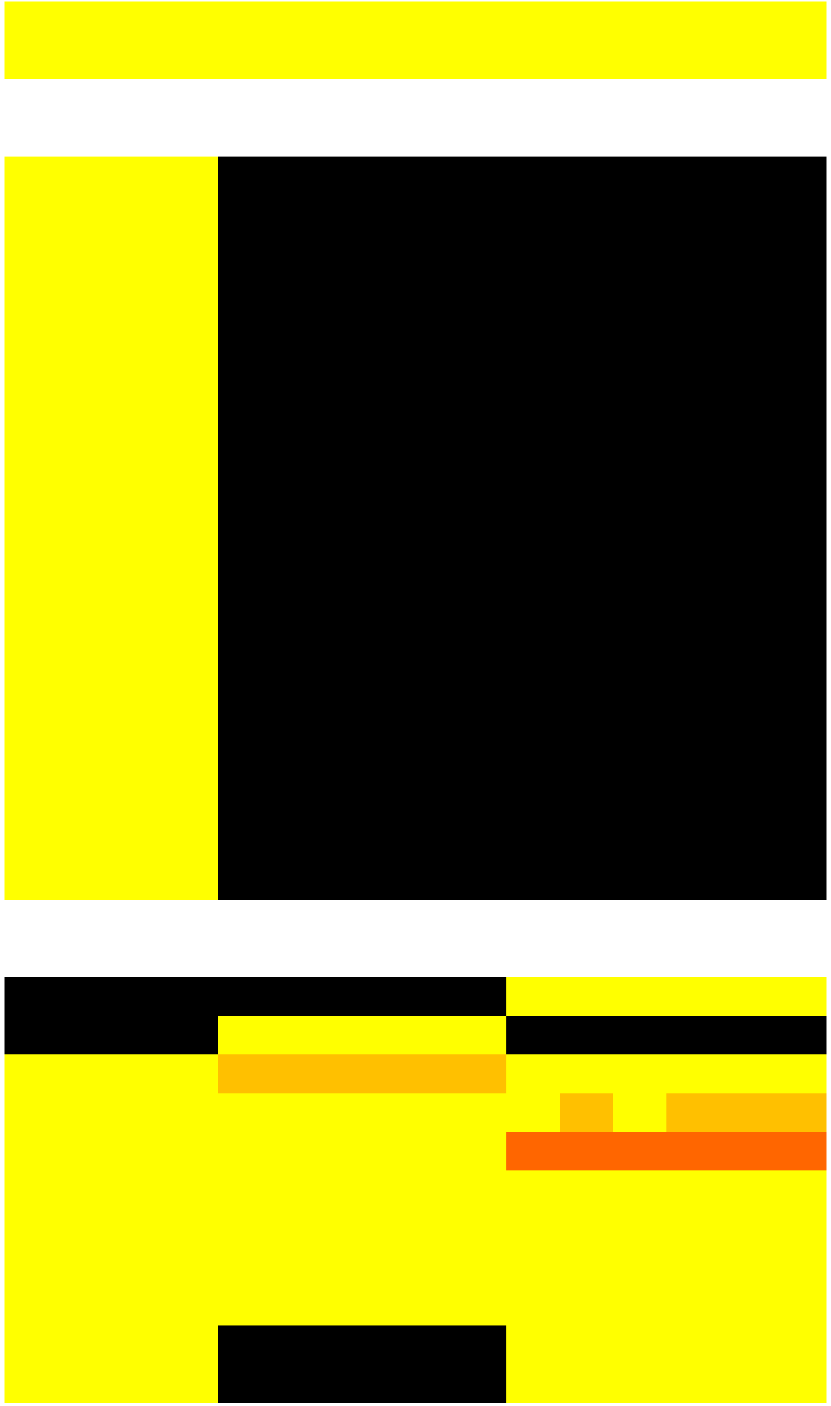

Prephenate and/or arogenate dehydrogenase, TyrA
Para-aminobenzoate synthase, TrpE
Anthranilate phosphoribosyltransferase, TrpD
Phosphoribosylanthranilate isomerase, TrpF
Indole-3-glycerol phosphate synthase, TrpC
Tryptophan synthase alpha chain, TrpA
Tryptophan synthase beta chain, TrpB

Cysteine and methionine biosynthesis

serine acetyltransferase (EC 2.3.1.30)
cysteine synthase (EC 2.5.1.47)
cystathionine gamma-lyase (EC 4.4.1.1)
cystathionine beta-synthase (EC 4.2.1.22)
S-adenosylhomocysteine nucleosidase (EC 3.2.2.9)
SAM-dependent methyltransferase
S-adenosylmethionine synthetase (EC 2.5.1.6)
homoserine O-acetyltransferase (EC 2.3.1.31)
homoserine dehydrogenase (EC 1.1.1.3)
O-succinylhomoserine sulfhydrylase (EC 2.5.1.48)
aspartate-semialdehyde dehydrogenase (EC 1.2.1.11)
aspartokinase (EC 2.7.2.4)
5-methyltetrahydrofolate--homocysteine methyltransferase (EC 2.1.1.13)
5-methyltetrahydropteroyltriglutamate--homocysteine methyltransferase (EC 2.1.1.14)

Indole-3-acetic acid biosynthesis

indolepyruvate ferredoxin oxidoreductase
indoleacetamide hydrolase , IamA, IamB (EC 3.5.1.-)
nitrilase, NitA (EC 3.5.5.1,7)
cobalt-containing nitrile hydratase (EC 4.2.1.84)
tryptophan 2-monooxygenase, IaaM

ROS tolerance

Superoxide dismutase [Cu-Zn] precursor
Superoxide dismutase [Fe]
Superoxide dismutase [Mn]
Rhodanese-related sulfurtransferase
Catalase-peroxidase

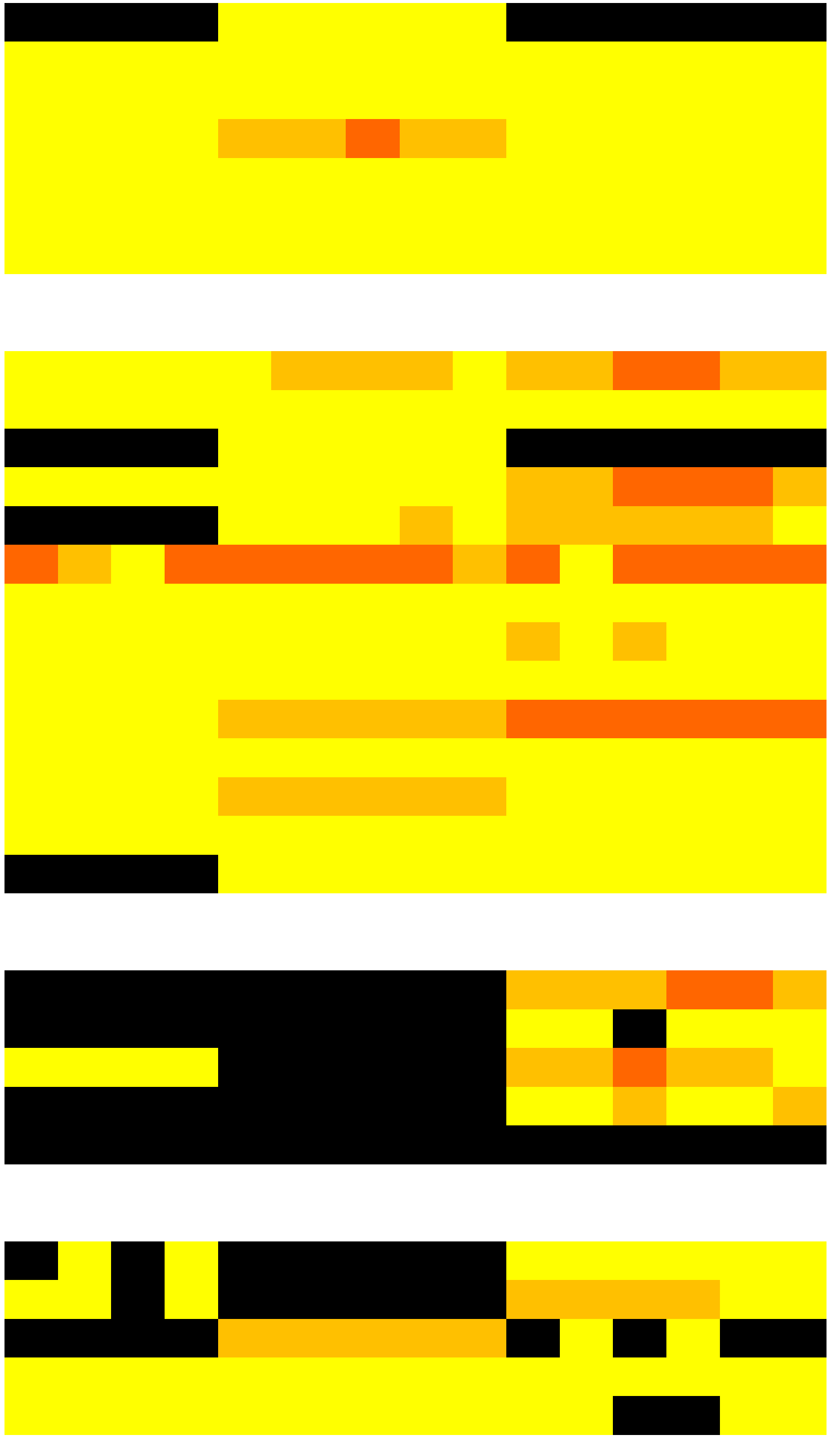

NTP pyrophosphohydrolases including oxidative damage repair enzymes

Cell wall-associated hydrolases (invasion-associated proteins)

Leucine-rich repeat proteins pfam13855, COG4886

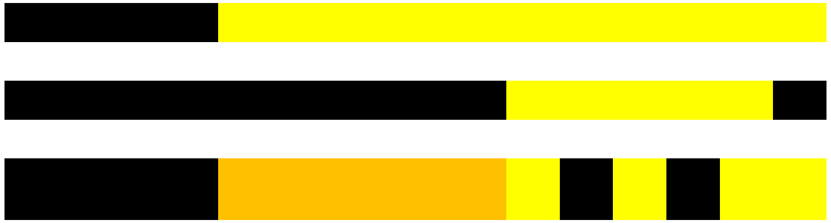

Supplement: Supplemental file 1 — Supplemental material. Download spectrum.00633-22-s0001.pdf, PDF file, 2.7 MB [file spectrum.00633-22-s0001.pdf]
